# The effects of perioperative dexmedetomidine infusion on hemodynamic stability during laparoscopic adrenalectomy for pheochromocytoma: a randomized study

**DOI:** 10.3389/fmed.2023.1276535

**Published:** 2023-11-02

**Authors:** Youngwon Kim, Young Chul Yoo, Na Young Kim, Hye Jung Shin, Ki Hong Kweon, Jiae Moon, Sang-Wook Kang

**Affiliations:** ^1^Department of Anesthesiology and Pain Medicine, Anesthesia and Pain Research Institute, Yonsei University College of Medicine, Seoul, Republic of Korea; ^2^Department of Research Affairs, Biostatistics Collaboration Unit, Yonsei University College of Medicine, Seoul, Republic of Korea; ^3^Department of Surgery, Yonsei University College of Medicine, Seoul, Republic of Korea

**Keywords:** pheochromocytoma, laparoscopic adrenalectomy, dexmedetomidine, hemodynamics, catecholamines

## Abstract

**Introduction:**

Pheochromocytoma is a rare catecholamine-producing neuroendocrine tumor originating from the adrenal medulla chromaffin cells. Hemodynamic instability can occur during the induction of anesthesia and surgical manipulation of the tumor. This study investigated the effects of intraoperative dexmedetomidine administration on hemodynamic stability in patients undergoing laparoscopic adrenalectomy for pheochromocytoma.

**Methods:**

Forty patients who underwent laparoscopic adrenalectomy for pheochromocytoma were randomly assigned to the dexmedetomidine (*n* = 20) or control (*n* = 20) group. The primary outcome of this study was intraoperative hemodynamic stability, and the secondary endpoint was the plasma catecholamine concentrations, specifically of epinephrine and norepinephrine.

**Results:**

The intraoperative maximum blood pressures were significantly lower in the dexmedetomidine group (control vs. dexmedetomidine group: 182 ± 31 vs. 161 ± 20, 102 ± 17 vs. 90 ± 10, and 128 ± 22 vs. 116 ± 12 [mean ± SD] mmHg and *p* = 0.020, 0.015, and 0.040 for systolic, diastolic, and mean blood pressure, respectively). The maximum heart rate during surgery was 108 ± 15 bpm in the control group and 95 ± 12 bpm in the dexmedetomidine group (*p* = 0.010). Other parameters of hemodynamic instability were comparable between both groups. Plasma catecholamine concentrations did not differ between the groups.

**Conclusion:**

Dexmedetomidine infusion following the induction of anesthesia at a rate of 0.5 μg/kg/h significantly attenuated the maximum intraoperative SBP, DBP, MBP, and HR, contributing to improved hemodynamic stability.

## 1. Introduction

Pheochromocytoma is a rare neuroendocrine tumor originating from chromaffin cells. The adrenal medulla consists of adrenergic and noradrenergic chromaffin cells, which generate and store catecholamines and release them into the bloodstream upon stimulation (1, 2). Pheochromocytoma causes secondary hypertension that can be cured through adrenalectomy. Due to the abrupt catecholamine release during the induction of anesthesia as well as surgical manipulation of the tumor, severe hemodynamic instabilities may occur, including hypertensive crises, cardiac arrhythmias, and cardiac ischemia (3–5). Conventional strategies to mitigate catecholamine-induced complications involve preoperative interventions using α-adrenergic antagonists (6). Nitric oxide modulators, including nitroprusside and nitroglycerin, along with esmolol (a beta-adrenergic antagonist), nicardipine (a calcium channel blocker), and magnesium, have been recognized as effective intraoperative treatments. These interventions are often used in combination, and are adjusted as necessary (7, 8). A physiological defense system exists to regulate potentially fatal hemodynamic fluctuations resulting from excessive catecholamine outflow. A key mechanism within this system is the inhibitory autoreceptor-mediated negative feedback, primarily mediated by the α2-adrenoreceptors, which operate similarly in adrenal medulla chromaffin cells (1, 9, 10).

Dexmedetomidine is a highly specific and selective α2-adrenoceptor agonist, exhibiting an α2 receptor affinity approximately eight times greater than clonidine (11). Furthermore, it has demonstrated sedative, analgesic, anxiolytic, and sympatholytic properties (12, 13). Dexmedetomidine administration by activating α2-adrenoceptors could potentially decrease the catecholamine availability, thereby mitigating the hypertensive response associated with pheochromocytoma (14, 15). However, there have been few case reports and no prospective studies regarding dexmedetomidine use in anesthesia for pheochromocytomas (15, 16). Therefore, we aimed to investigate the impact of dexmedetomidine administration on intraoperative hemodynamic stability in patients with pheochromocytoma.

## 2. Materials and methods

This study adhered to the Consolidated Standards of Reporting Trials (CONSORT) 2010 statement.

### 2.1. Patient population

Between December 2012 and March 2021, 42 patients were enrolled in this study between the ages of 20 and 70 years with an American Society of Anesthesiologists physical status classification I–III to undergo planned laparoscopic adrenalectomy for pheochromocytoma at Severance Hospital, Seoul, the Republic of Korea. All patients provided written informed consent before registration and received preoperative treatment with the orally administered phenoxybenzamine, a non-competitive α1 and α2 blocker for at least 3 weeks prior to the surgery. The following exclusion criteria were used for this study: (1) emergency operation; (2) reoperation; (3) combined surgery with other departments; (4) body mass index (BMI) >32 kg/m^2^; (5) history of arrhythmias (especially atrioventricular nodal block) and ventricular conduction abnormalities; (6) uncontrolled hypertension (diastolic blood pressure [DBP] >110 mmHg); (7) bradycardia (heart rate [HR] <40 bpm); (8) history of heart, hepatic, or renal failure, (9) history of cerebrovascular disease (cerebral hemorrhage, cerebral ischemia); (10) history of beta-blocker therapy; and (11) history of uncontrolled psychiatric disease.

### 2.2. Study design

According to a computer-generated randomization sequence, patients were randomly assigned to the dexmedetomidine group or control group. In the dexmedetomidine group, Precedex® (Hospira, Inc., Lake Forest, IL, USA) was immediately administered after the induction of anesthesia at a rate of 0.5 μg/kg/h and continued until the surgery was completed. In the control group, 0.9% normal saline infusion was immediately initiated after the induction of anesthesia at the same rate and continued until the end of the operation. Dexmedetomidine and 0.9% normal saline were transparent and indistinguishable upon visual inspection. The research nurse did not participate in the study besides managing and preparing the study medication. The nurse prepared sealed opaque envelopes indicating the group allocation according to the randomization sequence. Before general anesthesia induction, the nurse opened the envelope and prepared the study drug based on the group allocation, with 50 mL 0.9% normal saline for the control group and a mixture of 2 mL dexmedetomidine and 48 mL 0.9% normal saline (4 g/mL) for the dexmedetomidine group. The attending anesthesiologists, patients, and investigators were blinded to the group allocation.

### 2.3. Procedures

#### 2.3.1. Anesthesia

General anesthesia was performed after monitoring non-invasive blood pressure, electrocardiography, oxygen saturation, and bispectral index. Subsequently, a 0.1 mg of glycopyrrolate was administered intravenously for premedication. Anesthesia was induced by intravenous administration of propofol and remifentanil at 1–2 mg/kg and 0.05–0.1 μg/kg dose, respectively. Once the patient lost consciousness, tracheal intubation was facilitated using a 0.6 mg/kg dose of rocuronium. An invasive arterial catheter was inserted into the radial artery to monitor real-time blood pressure. Central venous catheterization was performed in all patients. Mechanical ventilation was maintained with 50% oxygen in the air and a respiratory rate of 10–15 breaths per minute. The tidal volume was set at 8 mL/kg based on the standard body weight, with a positive end-expiratory pressure of 5 cmH2O, end-tidal carbon dioxide maintained at 35–42 mmHg, and an inspiratory-expiratory ratio of 1:2. Study drug was administered immediately after the induction of anesthesia. If arrhythmia, including severe bradycardia (defined as HR <40 bpm), occurred, we discontinued the study drug immediately. Anesthesia was maintained using sevoflurane with the target Bispectral index™ (BIS, Medtronic Ltd., Minneapolis, MN, US) remaining between 40–60. Remifentanil was continuously infused at a rate of 0.03–0.1 μg/kg/min for maintenance of anesthesia. After the induction of anesthesia, the patient was placed in a prone position. To maintain the mean arterial pressure and heart rate at approximately 20% of the basal pre-anesthesia values during the operation, nitroprusside and esmolol, or norepinephrine, were used. During subcutaneous closure, fentanyl (Hana Pharm, Seoul, Korea) at a dose of 0.5 μg/kg and an antiemetic agent were administered intravenously.

#### 2.3.2. Laparoscopic adrenalectomy

Patients included in this study were approached using the preferred standard method of our institution: posterior retroperitoneoscopic adrenalectomy. After the induction of anesthesia, patients were placed in the prone jackknife position. The arms were flexed on arm boards with the elbows bent at a 90-degree angle, while the hips and knees were also bent at a 90-degree angle. The first incision was made 1.5 cm below the tip of the 12th rib, serving as the eventual location for the 12-mm trocar. The 10-mm trocar was placed approximately 2–3 cm medially from the first incision. The third 5-mm trocar was placed along the lowest margin of the 11th rib, approximately 4–5 cm lateral from the first incision. Subsequently, a 12-mm trocar was inserted through the first incision and CO_2_ was insufflated to a pressure of 18 mmHg, creating a pneumoretroperitoneum. The upper pole of the kidney was mobilized to expose the lower adrenal tumor portion. Subsequently, tumor dissection was performed from the medial to lateral direction, detaching the adrenal gland from the upper pole of the kidney. After careful medial dissection, the central adrenal vein was ligated. Furthermore, after complete adrenal gland dissection from the periadrenal fat, it was mobilized entirely for specimen removal.

### 2.4. Data collection

Hemodynamic instability was defined as a sudden increase or decrease in HR or blood pressure during surgery compared to the baseline hemodynamics. Hemodynamic stability was characterized by the HR and blood pressure remaining within an acceptable and relatively consistent range throughout the surgical procedure.

The primary endpoint of this study was intraoperative hemodynamic stability, which was assessed using the following parameters: (1) maximum blood pressure during surgery (systolic blood pressure [SBP], DBP, and mean blood pressure [MBP]); (2) duration of SBP increase by ≥30% from baseline (in minutes); (3) duration of SBP exceeding 200 mmHg; (4) maximum HR during surgery; (5) duration of HR exceeding 110 bpm during surgery; (6) duration of HR being <50 bpm; and (7) comparison of the quantity of vasoactive drugs (nitroprusside, esmolol, norepinephrine) used during surgery. Baseline blood pressure was defined as the blood pressure measured in the supine position before the induction of anesthesia in the operating room. These measures were used to evaluate and confirm hemodynamic stability during the surgical procedure.

As a secondary endpoint, the catecholamine secretion concentrations, specifically of epinephrine and norepinephrine, were measured at three different time points: immediately after the induction of anesthesia, during pheochromocytoma manipulation, and after surgery completion. At each time point, approximately 4 mL blood was collected using a heparin-containing vacutainer tube through an arterial catheter inserted during the induction of anesthesia. The collected blood was centrifuged within 60 min of collection at 1,800 g for 8 min at 4°C using a refrigerated centrifuge. After centrifugation, the plasma was obtained and transferred into labeled opaque polyethylene tubes, with each tube containing 0.8 mL plasma. Subsequently, the tubes were stored at temperatures below −70°C for freezing and sent for analysis. Plasma catecholamine concentrations were determined via a high-performance liquid chromatography (HPLC) analysis using Agilent 1,200 series (Agilent, Berkshire, UK) with electrochemical detection.

The following preoperative demographic characteristics were obtained: sex, age, BMI, American Society of Anesthesiologist physical status, 131I- or 123I- metaiodobenzylguanidine scintigraphy findings, preoperative hemodynamics (SBP, DBP, MBP, and HR), maximum tumor size, and tumor location as observed on computed tomography or magnetic resonance imaging. Surgical and perioperative characteristics, including surgical position; duration of anesthesia and operation; intraoperative blood loss and flood input; dose of nitroprusside, esmolol, and norepinephrine administered; number of patients who required rescue analgesics; and postoperative length of hospital stay were assessed. For preoperative and postoperative biochemical tests, we measured the 24-h urine and plasma catecholamine and metabolite concentrations, including metanephrine, normetanephrine, epinephrine, and norepinephrine.

### 2.5. Statistical analysis

Based on previous pheochromocytoma research (17), the maximum SBP recorded during surgery was 187 ± 30 (mean ± SD) mmHg. Considering a clinically significant reduction of 32 mmHg in the maximum SBP during surgery as an indicator of hemodynamic stability, the required sample size for intergroup comparison in this study was determined to be 21 patients per group, with an alpha of 0.05, 90% power, and an anticipated dropout rate of 10%.

For continuous variables, we conducted the Shapiro–Wilk test to assess the normality of the distribution. Normally distributed data were analyzed using the Student’s *t*-test. For non-normally distributed data, we performed the Mann–Whitney U test to investigate the differences between the groups. Categorical variables were analyzed using the chi-square test or Fisher’s exact test, and the results were presented as numbers (%). For repeatedly measured continuous variables, linear mixed models (LMMs) were employed. The mean profile plots were generated using the least-squares mean and standard error obtained from the LMM analysis. The adjusted *p*-values for comparisons between the two groups at each time point were calculated using Bonferroni correction to account for multiple comparisons. Statistical analyses were performed using SAS software (version 9.4; SAS Inc., Cary, NC, USA).

### 2.6. Ethics approval

This randomized study was approved by the Institutional Review Board and the Hospital Research Ethics Committee (Yonsei University Health System, Seoul, Korea; IRB Protocol No. 4–2012-0577). The trial was registered at ClinicalTrials.gov (identifier: NCT06037135).

## 3. Results

**Figure 1:**
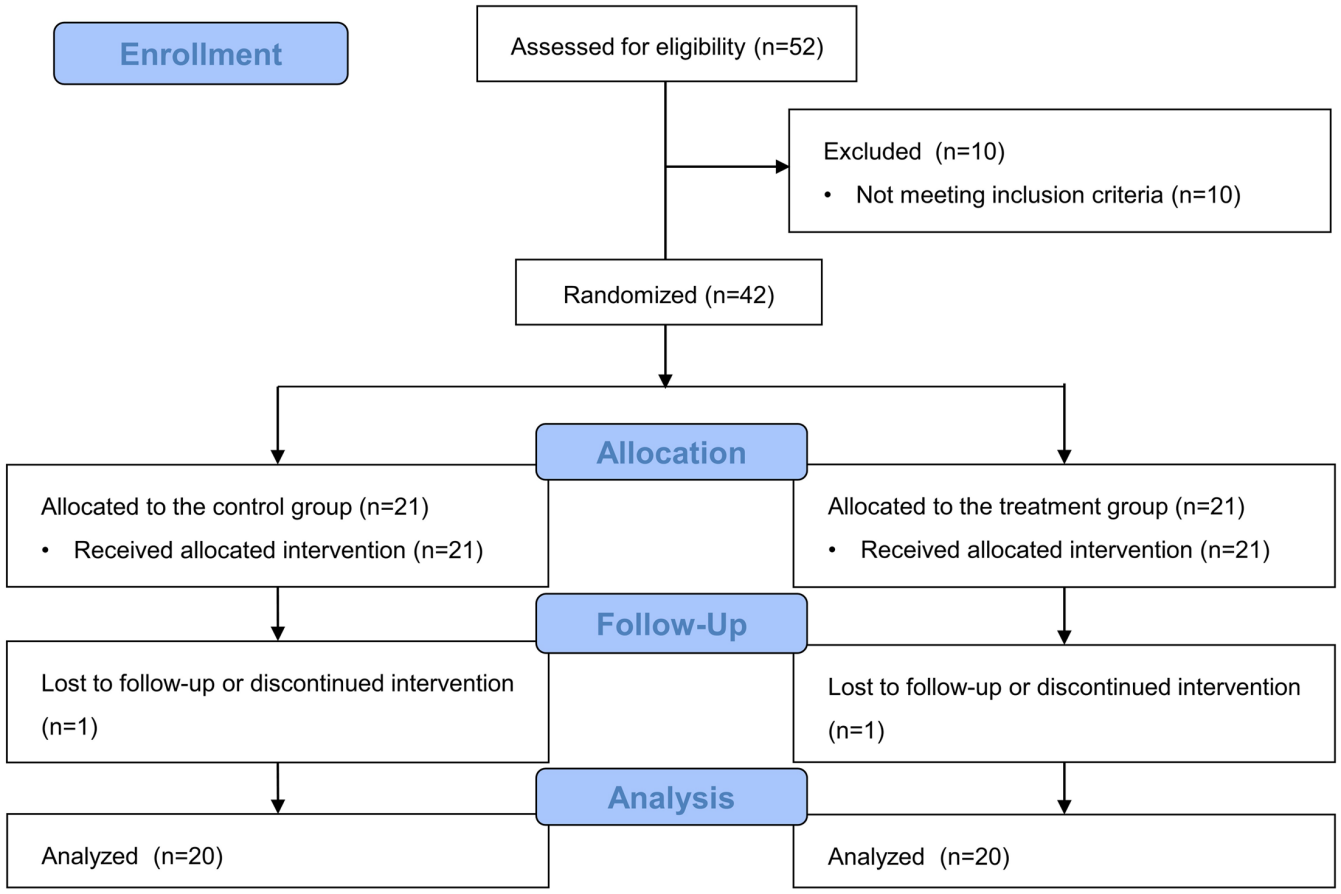
Flow diagram of the study.

In total, 52 patients who underwent laparoscopic adrenalectomy for pheochromocytoma were assessed for eligibility from December 2012 to March 2021, and 10 patients were excluded as they did not meet these criteria. The remaining 42 patients were randomly assigned to one of the two groups. In the control group, one patient changed their surgical position due to a failed approach. Furthermore, one patient from the dexmedetomidine group was excluded because of a different tumor characteristic. Therefore, 40 patients were included in the final analysis (Figure 1). No patient from either group developed arrhythmia, including severe bradycardia, during surgery.

Demographic and preoperative characteristics are described in Table 1. Patient age was significantly lower in the dexmedetomidine group than in the control group (*p* = 0.044). Other variables were comparable between the groups, including preoperative hemodynamics and maximum tumor size based on computed tomography or magnetic resonance imaging.

**Table 1:** Preoperative demographic characteristics.

	Control (*n* = 20)	DEX (*n* = 20)	*p* value
Female	8 (40%)	9 (45%)	0.749
Age, years	53 ± 12	44 ± 14	0.044*
BMI, kg/m^2^	23.7 ± 3.0	24.1 ± 4.4	0.746
ASA physical status			0.546
I	5 (25%)	7 (35%)	
II	8 (40%)	9 (45%)	
III	7 (35%)	4 (20%)	
MIBG scan	8 (40%)	6 (30%)	0.507
Preoperative hemodynamics			
Systolic BP, mmHg	132 ± 21	129 ± 17	0.617
Diastolic BP, mmHg	85 ± 15	78 ± 12	0.154
Mean arterial BP, mmHg	101 ± 16	95 ± 12	0.254
Heart rate, bpm	81 ± 12	82 ± 9	0.932
Maximum tumor size, cm	3.2 ± 1.3	3.5 ± 1.7	0.584
Tumor location			0.752
Right	10 (50%)	9 (45%)	
Left	10 (50%)	11 (55%)	

Data are presented as means ± standard deviations or number of patients (proportion). ^*^p < 0.05. DEX, dexmedetomidine; BMI, body mass index; ASA, American Society of Anesthesiologists; MIBG scan, ^131^I- or ^123^I- Metaiodobenzylguanidine scintigraphy; BP, blood pressure; bpm, beats per min.

Table 2 summarizes the surgical and perioperative parameters; the two groups had no significant differences. We used vasodilators for 55% of patients in the control group whereas for 30% of patients in the dexmedetomidine group. Vasopressors were used in 75 and 50% patients in the control and dexmedetomidine groups, respectively. The total amount of administered nitroprusside was significantly less in the dexmedetomidine group than in the control group. However, there was no statistically significant difference in the quantities of other vasoactive drugs used during surgery between the two groups.

**Table 2:** Surgical and perioperative characteristics.

	Control (*n* = 20)	DEX (*n* = 20)	*p* value
Surgical position			>0.999
Prone	17 (85%)	18 (90%)	
Lateral decubitus	3 (15%)	2 (10%)	
Anesthesia time, min	118 ± 33	126 ± 51	0.561
Operation time, min	78 ± 30	81 ± 47	0.794
Total fluid intake, mL	1,155 ± 440	1,113 ± 544	0.787
Blood loss, mL	10 (10, 30)	10 (10, 25)	0.476
Intraoperative vasodilators			
Total, *n*	11 (55%)	6 (30%)	0.110
Nitroprusside, *n*	11 (55%)	5 (25%)	0.053
Nitroprusside, μg	200 (0, 850)	0 (0, 75)	0.043*
Esmolol, *n*	3 (15%)	2 (10%)	>0.999
Esmolol, μg	3 ± 10	6 ± 18	0.554
Intraoperative vasopressors			
Total, *n*	15 (75%)	10 (50%)	0.103
Norepinephrine, *n*	15 (75%)	10 (50%)	0.103
Norepinephrine, μg	41.6 (4, 192.8)	8 (0, 112.8)	0.204
Rescue analgesics, POD1	7 (35%)	5 (25%)	0.490
Postoperative hospital days	3 ± 1	3 ± 1	0.846

Data are presented as means ± standard deviations, number of patients (proportion), or medians (first to third quartiles [Q1, Q3]). ^*^p < 0.05. DEX, dexmedetomidine; POD, postoperative day.

The variables related to hemodynamic stability during surgery are presented in Table 3. Significant differences between the two groups were observed in maximum SBP, DBP, MBP, and HR during surgery (*p* = 0.020, 0.015, 0.040, and 0.010, respectively). The maximal SBP was 182 ± 31 (mean ± SD) mmHg and 161 ± 20 mmHg in the control and dexmedetomidine groups, respectively. The maximum HR was 108 bpm in the control group while it was 95 bpm in dexmedetomidine group, respectively. The differences between the maximum and minimum SBP and HR, reflecting intraoperative hemodynamic fluctuations, were significantly greater in the control group (*p* = 0.015 and 0.007, respectively). The duration of time that SBP increased by >30% from baseline or exceeded 200 mmHg was comparable between the two groups. No significant differences were observed between the two groups regarding both the incidence and duration of HR above 110 bpm or below 50 bpm.

**Table 3:** Intraoperative hemodynamic parameters.

	Control (*n* = 20)	DEX (*n* = 20)	*p* value
Maximum			
Systolic BP, mmHg	182 ± 31	161 ± 20	0.020*
Diastolic BP, mmHg	102 ± 17	90 ± 10	0.015*
Mean arterial BP, mmHg	128 ± 22	116 ± 12	0.040*
HR, bpm	108 ± 15	95 ± 12	0.010*
Minimum			
Systolic BP, mmHg	85 ± 12	87 ± 7	0.493
Diastolic BP, mmHg	54 ± 8	49 ± 6	0.064
Mean arterial BP, mmHg	65 ± 9	62 ± 6	0.383
HR, bpm	61 ± 9	62 ± 10	0.925
Range^a^			
Systolic BP, mmHg	97 ± 32	74 ± 22	0.015*
Diastolic BP, mmHg	48 ± 18	41 ± 12	0.167
Mean arterial BP, mmHg	64 ± 22	54 ± 13	0.090
HR, bpm	46 ± 16	33 ± 10	0.007*
SBP >200 mmHg, *n*	4 (20%)	0 (0%)	0.106
SBP >200 mmHg, min	0.7 ± 1.9	0 ± 0	0.125
SBP > baseline +30%, *n*	10 (50%)	5 (26%)	0.129
SBP > baseline +30%, min	2.5 ± 3.2	1.7 ± 3.7	0.468
SBP > baseline – 30%, *n*	16 (80%)	13 (68%)	0.480
SBP > baseline – 30%, min	16.4 ± 21.6	7.3 ± 14.2	0.131
Heart rate > 110 bpm, *n*	6 (30%)	3 (16%)	0.451
Heart rate > 110 bpm, min	5.8 ± 21.1	3.7 ± 14.9	0.730
Heart rate < 50 bpm, *n*	2 (10%)	2 (10%)	>0.999
Heart rate < 50 bpm, min	0.2 ± 0.9	0.3 ± 1.0	0.812

Data are presented as means ± standard deviations or number of patients (proportion). ^*^p < 0.05. ^a^Calculated for each patient as the maximum value – minimum value. DEX, dexmedetomidine; BP, blood pressure; HR, heart rate; SBP, systolic blood pressure; bpm, beats per minute.

**Figure 2:**
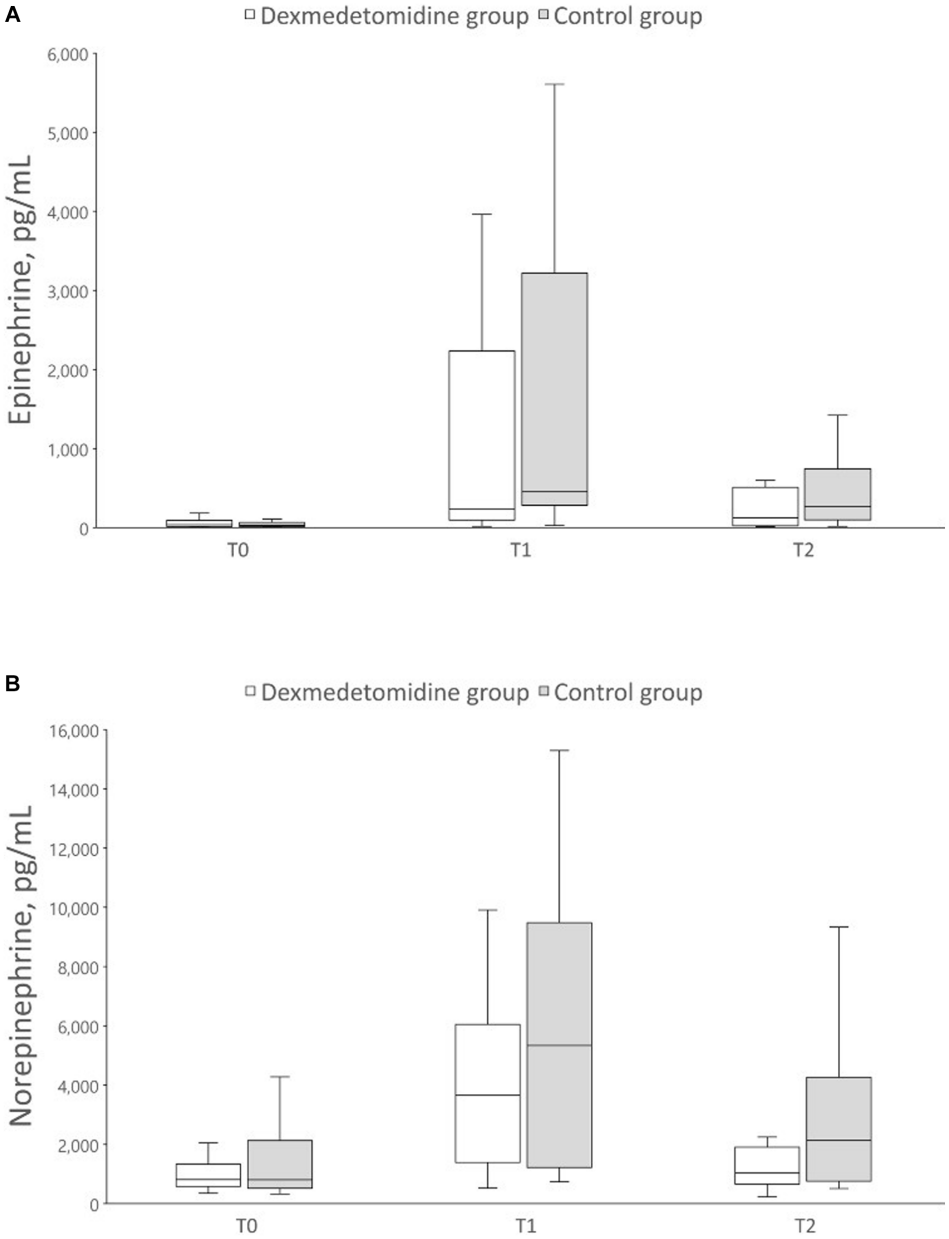
Box plot of perioperative catecholamine concentrations. The data are displayed as boxplot showing the first quartile (25%), the median (50%), and the third quartile (75%). The whiskers are displaying values within 1.5 times the interquartile range. **(A)** Epinephrine, **(B)** Norepinephrine. T0, preoperative; T1, during pheochromocytoma manipulation; T2, at the end of surgery.

Figure 2 summarizes the epinephrine and norepinephrine concentrations at each time point. During the manipulation of pheochromocytoma, epinephrine concentrations were 453.0 (281.5, 3217.0) [median (Q1, Q3)] and 23.5 (97.0, 2231.3) pg./mL in the control and dexmedetomidine groups, respectively. Additionally, norepinephrine concentrations were 536.0 (1215.5, 9479.5) and 3657.5 (1388.8, 6047.8) pg./mL in the control and dexmedetomidine groups, respectively. Catecholamine concentrations did not differ significantly between the groups at any time point.

The preoperative and postoperative biochemical test results, including urinary and plasma catecholamines and metabolite concentrations, are presented in Table 4. There were no differences between the two groups.

**Table 4:** Preoperative and postoperative biochemical tests.

	Control (*n* = 20)		DEX (*n* = 20)		*p* value
	Pre-op	Post-op	Pre-op	Post-op	
24-h Urine catecholamines					
MN, μg/day (52–341)	209 (107.2, 818.1)	39.1 (28.9, 101.6)	169.7 (69.2, 534.7)	67.6 (50.2, 88.5)	0.562
NMN, μg/day (88–444)	560.9 (351.6, 1612.3)	218.7 (179, 379.9)	637.3 (297.5, 1348.3)	213.3 (176.1, 258.5)	0.922
Epi, μg/day (0–17)	7.5 (3.8, 17.2)	2.2 (1, 4.3)	11.9 (4.2, 29)	2.6 (1.1, 4.2)	0.450
NE, μg/day (8–79)	84.1 (45.6, 107.2)	39.3 (24.3, 58.2)	79.2 (50.7, 214.7)	40.2 (31.4, 49.2)	0.305
Plasma catecholamines					
MN, nmol/L (0–0.5)	0.5 (0.29, 1.9)	0.2 (0.1, 0.2)	0.3 (0.15, 2.6)	0.1 (0.1, 0.2)	0.274
NMN, nmol/L (0–0.9)	3.1 (1.32, 8.1)	0.5 (0.4, 0.7)	2.1 (1.0, 5.1)	0.4 (0.4, 0.6)	0.632
Epi, pg./mL (0–110)	56.4 (39.9, 115.6)	35.9 (25.3, 48.3)	66 (50.3, 104.3)	28.1 (20, 33.4)	0.494
NE, pg./mL (7–750)	687.8 (293.7, 888.8)	439.7 (268.6, 642.5)	437 (321.8, 824.6)	319 (238, 397)	0.290

Data are presented as medians (first to third quartiles [Q1, Q3]). ^*^p < 0.05. DEX, dexmedetomidine; Pre-OP, pre-operative; Post-op, post-operative; MN, metanephrine; NMN, normetanephrine; Epi, epinephrine; NE, norepinephrine.

## 4. Discussion

In this prospective, randomized controlled study, we investigated whether dexmedetomidine administration during laparoscopic adrenalectomy for pheochromocytomas could improve hemodynamic stability. Dexmedetomidine infusion following the induction of anesthesia, at a rate of 0.5 μg/kg/h, significantly attenuated the maximum intraoperative SBP, DBP, MBP, and HR, contributing to improved hemodynamic stability. However, the two groups had no statistically significant difference in the actual intraoperative catecholamine secretion concentrations.

Pheochromocytoma is associated with substantial perioperative hemodynamic instability during surgery and requires careful anesthetic management. Preoperative alpha-blockade is routinely administered as a pretreatment strategy to mitigate these effects (3). However, despite appropriate preoperative preparation, perioperative hemodynamic instability is still reported at varying incidences, ranging from 8.7 to 69.3% (18–20). There is controversy regarding potential predictive factors for hemodynamic instability, including alpha-adrenergic receptor blocker administration duration, tumor type, and tumor size (20, 21). However, Tauzin-Fin et al. reported that hemodynamic instability during pheochromocytoma surgery is primarily determined by catecholamine secretion during surgical manipulation and is not predicted based on preoperative data (6). In our study, although all patients received a 3-week pretreatment with phenoxybenzamine according to the hospital protocol, hemodynamic instability occurred during tumor manipulation, requiring administration of low-dose esmolol, sodium nitroprusside, or nicardipine. We demonstrated that administration of dexmedetomidine in catecholamine surge situations effectively attenuated maximal blood pressure and successfully managed sudden hypertensive crises.

Hypertension and hemodynamic instability associated with pheochromocytoma are intricate mechanisms influenced by multiple factors, including the sympathetic nervous system and excessive catecholamine release into the bloodstream (22). Clonidine is a central α2-adrenoceptor agonist which decreases norepinephrine release and has been used perioperatively to attenuate hyperadrenergic states (23, 24); however, due to the properties of rebound hypertension and long half-life, the clinical application of clonidine has been limited (25). Dexmedetomidine, a highly selective α2-adrenoceptor agonist, specifically targets central receptors and provides sedation, analgesia, and centrally mediated sympatholytic effects (12, 13, 26). Talke et al. demonstrated that during emergence from anesthesia, dexmedetomidine attenuated the increase in HR and plasma norepinephrine concentrations in patients undergoing vascular surgery (14). Furthermore, they suggested that the hemodynamic effects of dexmedetomidine are partially mediated by its sympatholytic properties, with concurrent regulation of plasma norepinephrine concentrations. Wong et al. reported a case whereby dexmedetomidine was a valuable adjunctive anesthetic agent to maintain stable blood pressure and to prevent abrupt hypertensive crises in a patient undergoing adrenalectomy for a large pheochromocytoma with inferior vena cava invasion (27). Similar to previous studies, our study demonstrated that dexmedetomidine effectively suppressed the significant increase in maximum blood pressure during surgery. Consistent with these findings, the difference between the maximum and minimum SBP was less when using dexmedetomidine, indicating reduced intraoperative blood pressure fluctuation.

The younger age range of the patients in the dexmedetomidine group may have contributed to the superior maintenance of intraoperative blood pressure in this group. However, according to the findings of Urabe et al.’s meta-analysis, age is not significantly associated with perioperative hemodynamic instability during pheochromocytoma resection (28). Other studies identifying dexmedetomidine-associated hemodynamic instability demonstrated an increased incidence of dexmedetomidine-related hypotension with age, which is contrary to our findings (29, 30). The significant difference in age between the two groups might be due to the small sample size; thus, age-adjusted analysis could be helpful in interpreting the results.

The α2 adrenoceptor mediates negative feedback regulation of norepinephrine release at the synaptic terminal in various neuronal cells containing norepinephrine, including adrenal medulla chromaffin cells (1, 10). Previous studies have reported that dexmedetomidine decreases plasma norepinephrine concentration (13, 14, 16). However, we found no differences in catecholamine secretion between the two groups in this study. A low clinically approved dexmedetomidine dose was employed in this study to mitigate undesirable effects, including hypotension, significant bradycardia, and sinus arrest. Surgical manipulation of adrenal pheochromocytoma causes a substantial release of catecholamines during operation (6, 31). However, suppressing catecholamine release induced by tumor manipulation would be challenging with a low dose of dexmedetomidine. Therefore, during adrenalectomy for pheochromocytoma, where a significant catecholamine release is anticipated, a low dose of dexmedetomidine may be insufficient to adequately suppress catecholamine secretion. Although administration of dexmedetomidine did not directly affect the regulation of catecholamine secretion, it provided hemodynamic stability by reducing maximal blood pressure. Dexmedetomidine served as an effective adjunct to general anesthesia in patients with pheochromocytoma.

This study has several limitations. First, this study was a single-center study with a small sample size. Pheochromocytoma is a rare neuroendocrine tumor originating from adrenal medulla chromaffin cell, with reported incidence rates ranging from 0.04 to 0.95 cases per 100,000 individuals per year (32). It is noteworthy that the results of this study are clinically significant despite the relatively small sample size. Second, this study did not compare different dexmedetomidine doses but rather employed a single clinically approved low-dose infusion. Third, patients with cardiac, renal, and hepatic comorbidities and/or obesity were excluded from this study. It is difficult to generalize the results of our study because the pharmacokinetics of dexmedetomidine might be different in these patients. Additional research should be conducted to confirm the safety and efficiency of administrating dexmedetomidine during pheochromocytoma removal in such patients. Furthermore, this study only assessed intraoperative hemodynamic stability and catecholamine concentrations; long-term postoperative outcomes were not investigated. Further studies are required to obtain a more comprehensive understanding of dexmedetomidine’s role in pheochromocytoma surgery. However, this study is clinically meaningful as it significantly contributes to the knowledge regarding intraoperative dexmedetomidine use during laparoscopic adrenalectomy in patients with pheochromocytoma, particularly regarding its hemodynamic stability effect. Inspite of these limitations, our findings provide valuable insights that can serve as a foundation for future research. It would be necessary to conduct further research using a multicenter study design to clarify our study findings.

## 5. Conclusion

Intraoperative dexmedetomidine infusion at a dose of 0.5 μg/kg/h in patients undergoing laparoscopic adrenalectomy for pheochromocytoma demonstrated effective mitigation of the increase in maximum BP and HR. The results indicate a significant impact of dexmedetomidine on maintaining intraoperative hemodynamic stability. Dexmedetomidine could be considered as an effective adjuvant to general anesthesia in patients undergoing laparoscopic adrenalectomy for pheochromocytoma.

## Data availability statement

The raw data supporting the conclusions of this article will be made available by the authors, without undue reservation.

## Ethics statement

The studies involving humans were approved by Yonsei University Health System, Severance Hospital, Institutional Review Board. The studies were conducted in accordance with the local legislation and institutional requirements. The participants provided their written informed consent to participate in this study.

## Author contributions

YK: Conceptualization, Writing – original draft, Data curation, Formal analysis. YCY: Writing – review & editing, Conceptualization, Validation. NYK: Writing – review & editing, Validation. HJS: Formal analysis, Methodology, Software, Visualization, Writing – review & editing. KHK: Data curation, Investigation, Writing – review & editing. JM: Conceptualization, Project administration, Supervision, Writing – original draft, Writing – review & editing, Funding acquisition, Validation. S-WK: Project administration, Supervision, Writing – review & editing, Resources, Validation.

## Abbreviations

SBP: Systolic blood pressure
DBP: Diastolic blood pressure
MBP: Mean blood pressure
HR: Heart rate
BMI: Mass index
LMM: Linear mixed models
